# Review of brachytherapy clinical trials: a cross-sectional analysis of ClinicalTrials.gov

**DOI:** 10.1186/s13014-024-02415-8

**Published:** 2024-02-13

**Authors:** David Chen, Rod Parsa, Kabir Chauhan, Jelena Lukovic, Kathy Han, Amandeep Taggar, Srinivas Raman

**Affiliations:** 1 Princess Margaret Cancer Centre, Radiation Medicine Program, Toronto, ON Canada; 2 Temerty Faculty of Medicine, University of Toronto, Toronto, ON Canada; 3 Michael G. DeGroote School of Medicine, McMaster University, Hamilton, ON Canada; 4 Department of Radiation Oncology, University of Toronto, Toronto, ON Canada; 5 Odette Cancer Centre, Sunnybrook Health Sciences Centre, Toronto, ON Canada; 6 Department of Radiation Oncology, Princess Margaret Cancer Centre, 610 University Avenue, Toronto, ON M5G 2M9 Canada

## Abstract

**Introduction:**

Characterizing the landscape of clinical trials including brachytherapy can provide an overview of the current status and research trends which may guide further areas of investigation.

**Method:**

We queried 449,849 clinical trials from the ClinicalTrials.gov registry using brachytherapy-related keywords from 1980 to 2023, yielding 245 multi-arm and 201 single-arm, brachytherapy trials. Multi-arm and single-arm brachytherapy trials were compared using 12 trial protocol elements.

**Results:**

The number of trials including brachytherapy has increased over time, with over 60% of trials registered in 2010 onwards. The majority of clinical trials were Phase 2 or 3, evaluated both safety and efficacy, and were funded by academic sponsors. The most common tumor sites evaluated in brachytherapy clinical trials include prostate, cervix, liver, endometrium, and breast.

**Conclusion:**

There remains continued interest in clinical trials including brachytherapy focused on evaluation of novel delivery systems, treatment planning, and new indications. More brachytherapy clinical trials are needed to define the optimal clinical utilization and advance prospective research in this field.

## Introduction

Brachytherapy is a form of highly conformal radiotherapy that involves implantation of radiation sources into or near a target tumor using catheters [1]. Interstitial brachytherapy was first used in the treatment of prostate cancer (PCa) in the early twentieth century and later in the curative treatment of cervical cancer (CCa) [2]; since then, significant technological advances, such as image-guided planning, have led to improved outcomes in the management of multiple cancer types [3, 4]. The ability of brachytherapy to accurately deliver very high doses of radiation to the tumor while sparing surrounding tissues to minimize toxicity makes it particularly attractive for treatment of localized cancers [5, 6]. Given the excellent clinical outcomes with the integration of brachytherapy into the management pathway for PCa and CCa, the appropriate utilization of brachytherapy is strongly endorsed by most professional society guidelines [7, 8].

Despite these positive outcomes, there seems to be a decrease in brachytherapy use in modern radiation oncology for several cancer types [9–11]. The observed decrease may in part be attributed to lack of trainee exposure [12, 13], geographic variation in access to brachytherapy services [14], as well as low availability of required expertise and infrastructure [15]. Thus, the extent of interest in improving brachytherapy usage and advances remains unclear. Moreover, there exists a need to characterize the landscape of prospective research to identify future directions for modern brachytherapy.

Since the previously published reviews of clinical trials including brachytherapy in the mid 2010’s [16, 17], there have been several improvements to the tracking of implants, self-shielded applicators, image-guided application, and planning workflow of brachytherapy. Coupled with the application of machine learning to optimize treatment planning, these novel advances to the practice of brachytherapy shows great promise to increase the broad utility and ease of practicing brachytherapy in modern radiation oncology [18]. The continued development of brachytherapy and external beam radiotherapy (EBRT) technologies coupled with evolution of systemic therapies may change indications and facilitate new research directions across various cancers.

As a contemporary follow up study to previous reviews of brachytherapy clinical trials, we analyzed the features of radiation oncology clinical trials including brachytherapy sourced from the clinical trial registry, ClinicalTrials.gov, to date to characterize the landscape of prospective brachytherapy research over time.

## Methods

We queried ClinicalTrials.gov, the largest standalone registry of global clinical trials, on April 25, 2023 using the MeSH and non-MeSH keywords “radiotherapy”, “radiation therapy”, “targeted radiation therapy”, “radiation”, “radiation treatment”, and “targeted radiotherapy, yielding 21,474 radiotherapy-related trials out of 449,849 clinical trials in total. Next, we queried this subset with the MeSH and non-MeSH keywords “brachytherapy”, “radioisotope brachytherapy”, “curietherapy”, “implant radiotherapy”, “interstitial radiotherapy”, “intracavity radiotherapy”, “radioisotope plaque therapy”, “radioisotope brachytherapy”, and “surface brachytherapy”, yielding 2,126 brachytherapy-related trials. We only included trials that were classified as completed or ongoing (excluding 258 trials) and interventional (excluding 329 trials which were observational), for a total of 1,539 interventional (non-observational) clinical trials including brachytherapy that were completed or ongoing based on the brachytherapy-related keyword search. In this study, we chose to include trials on clinicaltrials.gov which were classified as “brachytherapy” but referring more broadly to internal radiation treatment including selective internal radiation therapy (SIRT).

**Fig. 1:**
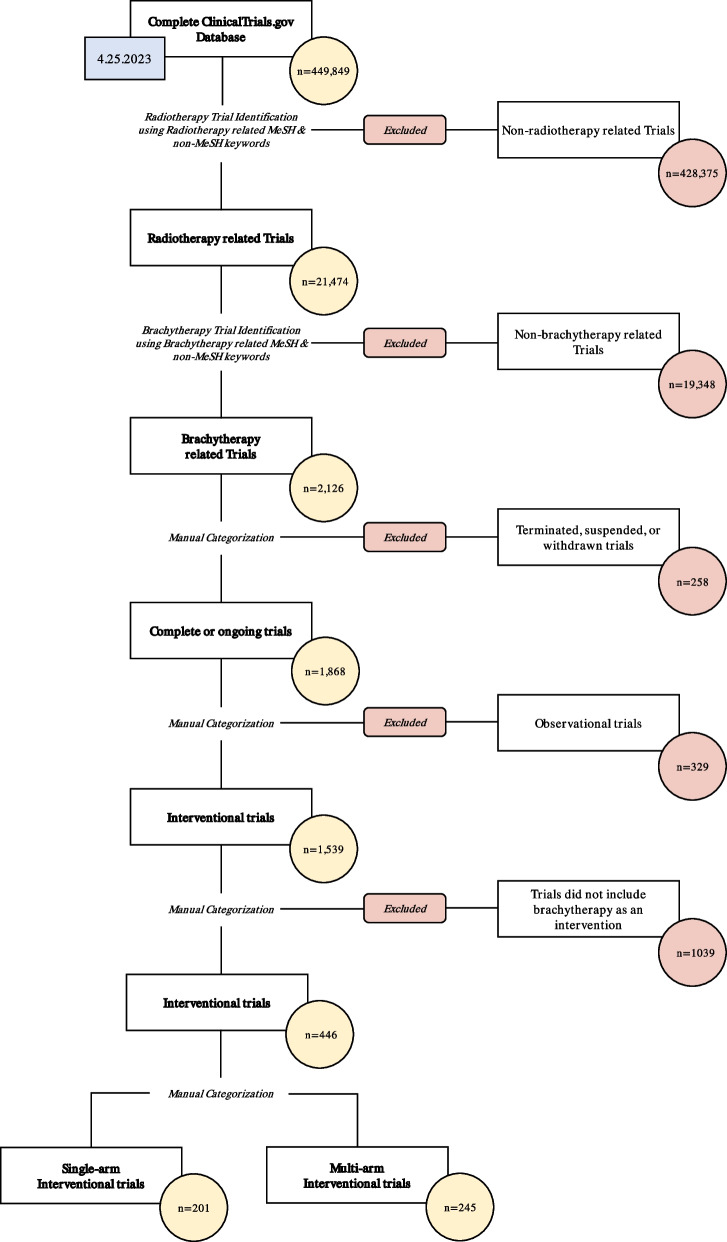
Search and filtering strategy used to select trials including brachytherapy from the ClinicalTrials.gov registry

To examine the characteristic differences between single-arm trials primarily focused on testing safety and multi-arm trials primarily focused on testing efficacy of brachytherapy treatment, we split the collected trials into either single-arm trials or multi-arm trials based on the number of treatment arms of the trial. Out of the 1,539 interventional, completed or ongoing clinical trials, we then conducted a manual screen to confirm that each trial did indeed include brachytherapy in at least one treatment arm, yielding 201 single-arm trials and 245 multi-arm trials. We followed the same methodology as Cihoric et al. [16] to collect the set of trial features for the 201 single-arm clinical trials and 245 multi-arm clinical trials included in this study (Fig. 1). Trial characteristics with no available data were coded as “Data not available” for transparency.

For each clinical trial feature, we report the count of trials and proportion out of the total number of trials in the trial population as a percentage.

## Results

**Fig. 2:**
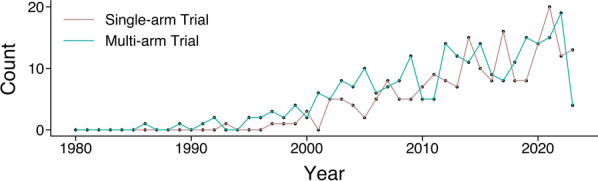
Number of multi-arm and single-arm trials including brachytherapy from January 1980 to April 2023

Our study included 446 clinical trials, including 245 multi-arm trials and 201 single-arm trials (Table 1). The number of both multi-arm and single-arm brachytherapy trials has increased over time since the 1980s, with over 60% of all trials having been initiated from 2010 onwards (Fig. 2).

**Table 1:** Characteristics of all brachytherapy trials (n = 446)

Category	Type	Count	Proportion
Type of brachytherapy	HDR	169	0.379
	LDR	138	0.309
	Unclear	104	0.233
	HDR/LDR	28	0.063
	HDR/PDR	5	0.011
	PDR	2	0.004
Number of trial arms	Single arm	201	0.451
	Multiple arms	245	0.549
Protocol initiator	Academic	347	0.778
	Collaborative groups	35	0.078
	Industry	55	0.123
	NIH	9	0.02
Source of funding	Academic	279	0.626
	NIH	59	0.132
	Industry	93	0.209
	Collaborative groups	9	0.02
	Public–private partnership	6	0.013
Organ	Prostate	129	0.289
	Cervix	82	0.184
	Liver	41	0.092
	Other	31	0.07
	Endometrium	19	0.043
	Breast	37	0.083
	Esophagus	12	0.027
	Rectum	13	0.029
	Vaginal	8	0.018
	Uterus	13	0.029
	Brain	14	0.031
	Pancreas	12	0.027
	Eye	5	0.011
	Skin	13	0.029
	Lung	7	0.016
	Salivary gland	1	0.002
	Head and neck	8	0.018
	Kidney	1	0.002
Malignancy	Yes	437	0.98
	No	9	0.02
Country	United States	152	0.341
	International	39	0.087
	Canada	61	0.137
	China	41	0.092
	Data not available	24	0.054
	France	25	0.056
	India	11	0.025
	United Kingdom	10	0.022
	Netherlands	6	0.013
	Poland	6	0.013
	Germany	11	0.025
	Brazil	5	0.011
	Russian Federation	3	0.007
	Italy	6	0.013
	Bangladesh	1	0.002
	Thailand	1	0.002
	Saudi Arabia	1	0.002
	Czechia	1	0.002
	Korea, Republic of	1	0.002
	Norway	1	0.002
	Singapore	2	0.004
	Japan	2	0.004
	Iran, Islamic Republic of	1	0.002
	Azerbaijan	1	0.002
	South Africa	1	0.002
	Indonesia	1	0.002
	Hungary	1	0.002
	Mexico	1	0.002
	Finland	2	0.004
	Austria	3	0.007
	Israel	9	0.02
	Spain	4	0.009
	Australia	3	0.007
	Denmark	2	0.004
	New Zealand	1	0.002
	Hong Kong	1	0.002
	Egypt	1	0.002
	Argentina	1	0.002
	Taiwan	1	0.002
	Slovenia	1	0.002
	Cyprus	1	0.002
Trial phase	Phase 3	96	0.215
	Phase 2	129	0.289
	Data not available	122	0.274
	Phase 4	16	0.036
	Phase 1	37	0.083
	Phase 2/phase 3	9	0.02
	Phase 1/phase 2	27	0.061
	Early phase 1	10	0.022
Endpoint classification	Safety/efficacy	296	0.664
	Efficacy	74	0.166
	Other	31	0.07
	Safety	39	0.087
	Data not available	6	0.013
Primary purpose	Treatment	407	0.913
	Supportive care	14	0.031
	Other	8	0.018
	Prevention	5	0.011
	Screening	2	0.004
	Health services research	3	0.007
	Device feasibility	3	0.007
	Diagnostic	3	0.007
	Data not available	1	0.002
Status	Completed	159	0.357
	Unknown status	85	0.191
	Recruiting	120	0.269
	Active, not recruiting	54	0.121
	Not yet recruiting	24	0.054
	Suspended	3	0.007
	Enrolling by invitation	1	0.002

**Fig. 3:**
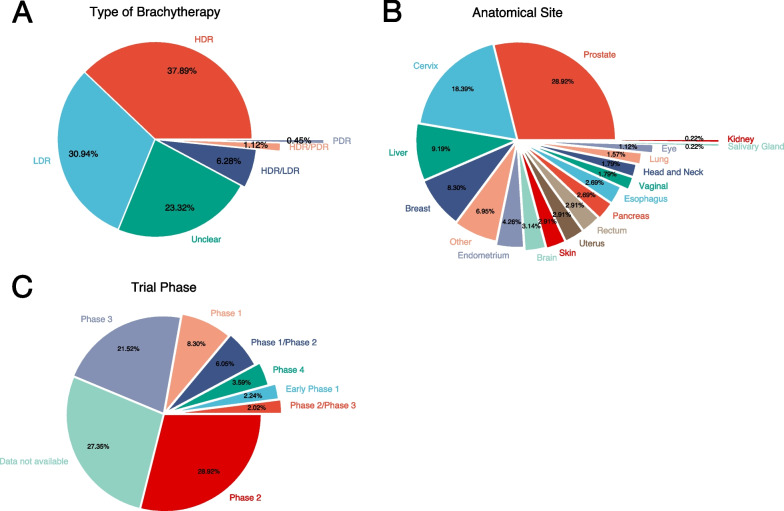
Proportion of all brachytherapy trials by **A** type of brachytherapy, **B** primary anatomical site, and **C** trial phase

With respect to the type of brachytherapy, trials most commonly involved high-dose-rate (HDR; 38%) followed by low-dose-rate (LDR; 31%) (Fig. 3A). The most common primary site of treatment was the prostate in 29% of trials. Other common primary anatomical sites included the cervix (18%), liver (9%), and breast (8%) (Fig. 3B). The overwhelming majority (98%) of brachytherapy trials were used in trial applications involving malignant disease, with the remaining 2% testing brachytherapy in benign conditions. Among all trials, the most common trial phase was Phase 2 (29%), followed by Phase 3 (22%) (Fig. 3C). Thus, it follows that Safety/Efficacy (66%) and Efficacy alone (17%) were the two most common endpoints of all trials. Phase 4 trials evaluating long-term safety and efficacy were relatively rare (4%).

**Fig. 4:**
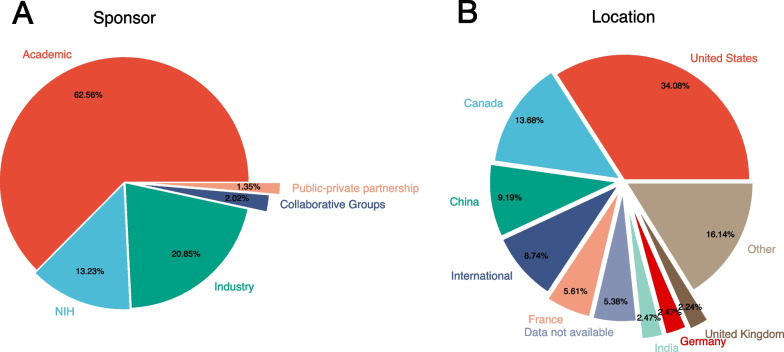
Proportion of all brachytherapy trials by **A** financial sponsor and **B** geographical location

Academic entities most frequently initiated 78% of trials, while also funding 63% of trials (Fig. 4A). Funding from the National Institute of Health (NIH) and industry made up 13% and 20% of trials respectively. The most common location of brachytherapy trials was in the United States (34%), followed by Canada (14%), China (9%), and collaborative, multinational trials (9%) (Fig. 4B).

Participant allocation in multi-arm brachytherapy trials were commonly randomized in 79% of multi-arm trials, with the parallel assignment being used as the most common intervention design in 81% of trials (Table 2). Other trial intervention designs used in multi-arm trials include single group assignment (7%), cross-over assignment (2%), sequential assignment (2%), and factorial assignment (1%).

**Table 2:** Randomization and participant allocation characteristics of multi-arm brachytherapy trials (n = 245)

Category	Type	Count	Proportion
Allocation	Randomized	194	0.7918
	Non-randomized	51	0.2082
Intervention model	Parallel assignment	198	0.8082
	Data not available	20	0.0816
	Single group assignment	17	0.0694
	Crossover assignment	4	0.0163
	Sequential assignment	4	0.0163
	Factorial assignment	2	0.0082

To highlight new directions of brachytherapy research, we observed that single-arm brachytherapy trials evaluated the performance of novel applications and new indications in 52% of single-arm trials, followed by techniques and equipment in 31% of single-arm trials (Table 3). A minority of single-arm trials investigated brachytherapy coupled with pharmacological intervention (7%), dose fractionation (7%), brachytherapy coupled with hyperthermia (2%), and brachytherapy coupled with photodynamic therapy (1%).

**Table 3:** Brachytherapy intervention applications of single-arm brachytherapy trials (n = 201)

Category	Type	Count	Proportion
Intervention	Novel application/new indication	105	0.522388
	Technique/equipment	62	0.308458
	Brachy + medication	15	0.074627
	Dose fractionation	14	0.069652
	Brachytherapy + hyperthermia	4	0.0199
	Brachy + photodynamic therapy	1	0.004975

## Discussion

In this study, we report the characteristics of 446 brachytherapy clinical trials initiated between January 1, 1980 to April 25, 2023 and registered on ClinicalTrials.gov. Despite previous reports of the decline of prospective brachytherapy clinical trials in the mid-2010s due to the need for experienced personnel and specialized equipment [16, 17], we observed that the count of brachytherapy trials increased over time. This finding suggests that there remains continued interest in the initiation of both exploratory, single-arm trials and comparative, multi-arm trials. This interest in brachytherapy, however, may be confounded by an overall increase in interest in radiotherapy as a treatment modality. In fact, subsequent reports observed increasing rates of brachytherapy use for CCa in the United States, increasing rates of brachytherapy use for PCa in Ontario, Canada [19], and scientometric trends that highlight broad interest in the development of automation and artificial intelligence in brachytherapy [20]. Taken together, our observation of increasing brachytherapy clinical trials over the study time period aligns with recent reports that observed an increase in brachytherapy utilization since the mid-2010s.

The majority of brachytherapy trials (63%) were funded by academic sponsors, followed by industry sponsors (21%) and the NIH (13%). Compared to a previous study of brachytherapy trials registered in ClinicalTrials.gov in 2016 that reported 89.4% of all brachytherapy trials funded by academic sponsors, our results suggest a comparative increase in the proportion of trials funded by non-academic sources. This trend of increasing brachytherapy interest may in part be attributed to social media promotion [21], modern training modalities using novel learning technologies [22, 23], and concerted efforts by the American Brachytherapy Society (ABS) to develop a national, longitudinal brachytherapy curriculum with a renewing certification process [24]. Although brachytherapy has a long history of evidence-based indications in many tumor sites, including cervix, endometrium, prostate, and breast, there remains interest in expansion of brachytherapy to novel indications in the esophagus, liver, rectum, brain, and pancreas based on our review of brachytherapy clinical trials [1]. Lastly, the emergence of brachytherapy clinical trials across multi-national sites is a promising sign of brachytherapy interest that can help spur increased knowledge-sharing and training in novel advances of brachytherapy worldwide.

Our study is limited to the review of brachytherapy clinical trials registered in ClinicalTrials.gov. Despite being the largest standalone registry of global clinical trials, there may exist clinical trials that are not compliant with mandatory registration in ClinicalTrials.gov or were initiated before 2007 when registration became mandatory. In addition, some clinical trials did not report all trial characteristics, and may not have up to date information about their current status.

## Gynecology

### Cervical cancer

Brachytherapy is an essential component of definitive management of locally advanced (FIGO stage IB3-IVA) cervical cancer that is associated with a survival benefit [10, 25, 26]. ASTRO guidelines for CCa strongly recommend integration of brachytherapy for intact CCa and conditionally recommend post-operative brachytherapy boost in the presence of positive margins [7]. While intracavitary applicators have historically been the dominant technique for CCa brachytherapy delivery, interstitial needles can improve dosimetry especially for larger tumors, lower vaginal involvement, intact uterus, lateral extension of disease, and ill-fitting intracavitary applicators [27, 28]. The EMBRACE II trial has several aims, including increased use of combined intracavitary/interstitial technique.

Several recent trials are focused on testing novel imaging techniques to improve precision or patient experience during brachytherapy, such as catheter navigation (NCT03781271) and MR-guided tracking (NCT03277469). Among single-arm trials, investigations have been aimed at fine-tuning the characterization, planning, and precise treatment of CCa using MRI and PET/MRI image-guided approaches (NCT03617133, NCT03655977), or tested innovative additions to the standard of care (NCT03308604, NCT03249519). Overall, the continued improvement of brachytherapy techniques, in addition to advances in chemotherapy and immunotherapy, may lead to improvements in both clinical outcomes and patient experience during the management of CCa.

### Endometrial cancer

ECa is treated surgically in over 90% of cases with hysterectomy, bilateral salpingo-oophorectomy (TH-BSO) ± lymph node sampling. Adjuvant vaginal brachytherapy is recommended for high-intermediate risk ECa [29]. Patients with isolated vaginal recurrence of ECa with no prior history of radiation treatment are salvaged with EBRT followed by a brachytherapy boost [30].

Recent trials focused on ECa have incorporated molecular classification [31]. Specifically, the PORTEC-4a trial (NCT03469674) uses integrated clinicopathological and molecular risk profiles to determine whether stage I-II high-intermediate risk ECa patients should receive no adjuvant therapy, vaginal brachytherapy, or EBRT based on their molecular-integrated risk profile. The ongoing international RAINBO trial (NCT05255653) investigates four molecular class-directed adjuvant treatment strategies [32]. For example, the RAINBO *POLE*mut-BLUE phase II trial evaluates the safety of de-escalation of adjuvant therapy (including brachytherapy) in patients with stage I-III *POLE*mut endometrial cancer. Recent analysis of PORTEC-1 and PORTEC-2 trials shows that molecular classification of ECa predicts response to radiotherapy in stage I endometrioid ECa [33]. Overall, advances in ECa have focused on personalized care by molecular subtype. Further research is needed to confirm the role of brachytherapy across molecular subgroups.

## Breast

The ABS recommends interstitial brachytherapy and intensity-modulated accelerated partial breast irradiation (APBI) as treatment options for patients with invasive cancers or ductal carcinoma in situ with no lymph node involvement and negative margins [34]. Interstitial multicatheter brachytherapy is an effective technique to deliver APBI for early-stage breast cancer (BCa) patients [35].

Many recent BCa trials have focused on testing the safety and efficacy of APBI as adjuvant treatment among patients with low-risk, early-stage BCa. There is interest in evaluating an interstitial brachytherapy boost following breast-conserving surgery and EBRT [36, 37]. The preliminary results of an NSABP-initiated randomized phase 3 trial (n = 4216) evaluating the efficacy of partial breast irradiation compared with whole breast radiation therapy showed that APBI with interstitial brachytherapy was not inferior in quality of life compared to whole-breast irradiation [38]. In the case of ipsilateral breast tumor recurrence, multicatheter brachytherapy coupled with lumpectomy can prevent future local recurrence with overall survival comparable to salvage mastectomy, and good cosmetic results reported in 85% of trial participants (n = 217) [39]. In a 10-year non-inferiority follow up study (n = 1328), early breast cancer patients who underwent post-breast conserving surgery APBI had comparable treatment efficacy compared to whole-breast irradiation and fewer late side effects [40].

The direct evaluation of novel dosing regimens and fractionations remains an area of active research interest. We highlight a phase 2 trial of partial breast brachytherapy for patients with early stage BCa resected by lumpectomy (NCT01185145), a phase 2 trial of a novel 3-fraction daily dosing regimen for APBI (NCT02453737), and a phase 2 trial of accelerated radiotherapy delivered to the lumpectomy cavity as a single dose brachytherapy treatment (NCT00185744). Further research is needed to determine whether intracavitary brachytherapy has superior clinical outcomes compared to EBRT, either as a primary treatment or adjuvant therapy to first-line EBRT and chemotherapy, in high-risk, late-stage BCa. The only application of novel technologies for balloon breast brachytherapy was the use of the MammoSite Multi-Lumen targeted radiation therapy system (NCT01448447, NCT01185145, NCT00103181), and future research may further improve the clinical and cosmetic outcomes associated with novel delivery systems.

## Prostate

The American Society for Clinical Oncology and Cancer Care Ontario guideline update jointly recommends LDR brachytherapy, EBRT, or radical prostatectomy for favourable-risk PCa patients. For unfavourable-risk PCa patients, EBRT with androgen-deprivation therapy and potentially LDR/HDR brachytherapy boost is recommended [8]. Historically, interstitial brachytherapy has been a cornerstone in the treatment of PCa [2], and several studies have demonstrated the potential for improved cancer control in the monotherapy and boost setting [41, 42]. There are several trials aimed at improving the implantation technique, including the FAST trial which has studied texture-coated iodine-125 (I-125) seeds to limit post-implant displacement and migration (NCT01174017) and the J0511 trial investigating robot-guided radioactive seed implantation (NCT00381966).

Partial and focal brachytherapy is an area of growing interest as a treatment and there is a prevalence of trials exploring this modality, based on the favourable oncological outcomes and toxicity profile associated with this approach in the definitive and recurrent setting [43, 44]. Both Loyola University and Sunnybrook Health Sciences Centre have focused recent efforts on focal salvage HDR brachytherapy for locally recurrent PCa (NCT03312972, NCT01583920). LDR focal therapy is also an active area of exploration, with trials exploring the value of focal hemi-ablative treatment via transperineal template-guidance and multiparametric MRI (NCT02643511, NCT01830166).

Finally, we observe an interest in the combination of brachytherapy and various EBRT techniques in the treatment of PCa. The BRAchySABR trial, for example, has investigated HDR brachytherapy in combination with stereotactic body radiotherapy (NCT04523896), and similar trials have been noted at a number of international sites (NCT04945642, NCT05754580, NCT04236752, NCT02280356, NCT01655836). We note that HDR brachytherapy boost is a consistent treatment modality in several trials investigating fraction schema for external beam radiotherapy (NCT05820633, NCT04861415, NCT04100174, NCT02303327, NCT04861415).

## Gastrointestinal

### Esophageal cancer

Brachytherapy remains underutilized and under-explored in the management of esophageal cancer (ECa) [45]. Progressive and malignant dysphagia is a common presenting symptom and may be managed using expanding metallic stents to improve quality of life [46]. We note some interest in investigating brachytherapy in the management of dysphagia, particularly in the post-stent placement setting. These studies aimed to optimize management of dysphagia by comparatively examining the addition of a single dose of HDR brachytherapy to stent insertion (NCT01366833), similar to the BRASTEGAC trial (NCT01786278). Lastly, we highlight a phase III trial comparing chemoradiotherapy with or without the addition of iridium-192 brachytherapy (NCT00002884). Overall, we note a limited number of studies for the value of brachytherapy in the management of ECa.

### Liver cancer

Selective internal radiation therapy (SIRT) or radioembolization, considered a type of brachytherapy, can be used to treat unresectable or inoperable liver cancer (LCa) [47]. Due to the hypervascularity of the liver parenchyma, SIRT can be used to selectively deliver targeted doses of radioactive sources, such as yttrium-90 microspheres, through the liver’s blood supply to treat malignancies [47]. Further research is needed to compare the safety and efficacy of brachytherapy to local ablative EBRT and systemic therapies in patients with non-resectable HCC and intrahepatic cholangiocarcinoma [48]. Although SIRT remains a primary area of research for liver malignancy indications, evaluations of HDR brachytherapy have previously been reported to be an effective treatment of liver metastases with good local control and low toxicity [49, 50]. Long-term follow-up studies of LDR (n = 64) and HDR (n = 75) [51] brachytherapy to treat liver malignancies concluded that brachytherapy is an effective treatment option for unresectable primary and metastatic tumors, with one year LDR local control rates of 44% and HDR local control rates of 48–94% based on tumor size.

To date, SIRT remains the most prominent focus of clinical trials in LCa. Active trials are investigating the safety and efficacy of SIRT compared to tremelimumab and durvalumab immunotherapies (NCT05701488) for resectable and locally advanced HCC. In addition, trials studying the optimization of SIRT dosimetry (NCT02582034, NCT05227482) may highlight new methods to improve clinical outcomes while reducing adverse events associated with brachytherapy. For patients with unresectable biliary tract cancer (intrahepatic or extrahepatic cholangiocarcinoma), intraluminal brachytherapy stents for irradiation treatments remains one area of continued research (NCT02238613). Lastly, there remains ongoing research to design radiation delivery microspheres that can be visualized via fluoroscopy, X-ray, and CT imaging modalities to improve procedural accuracy (NCT04926376).

## Other select malignant and non-malignant diseases

Among other malignant diseases, we highlight brain and pancreatic cancers as sites with emerging activity that were identified in this study of the ClinicalTrials.gov registry. In 2020, GT Medical Technologies sponsored a trial investigating intracavitary Cs-131 brachytherapy during craniotomy (NCT04690348). Later in 2021, a trial at Qingdao University focused on novel regimens for glioblastoma, comparing I-125 brachytherapy together with chemotherapy to surgical resection and post-surgical concomitant chemoradiotherapy. The efficacy of I-125 is being further explored in relation to locally advanced pancreatic cancers, with China leading two trials using 3D-printed template-assisted CT implantation. The first evaluates the safety and efficacy of the 3D-printed templates (NCT03882866), while the second compares the I-125 treatment to stereotactic radiotherapy (NCT03964064). The results from these trials may support a growing body of evidence supporting the use of interstitial brachytherapy in advanced pancreatic cancers [52].

The usage of I-125 was revisited in the context of malignant central airway stenosis, with researchers assessing the efficacy of I-125-loaded metal stents (NCT03944408), building upon prior monocentric control studies [53]. Expanding on recent trials of SIRT indicated for liver malignancies, Sirtex Medical recently completed phase 3 trials assessing the efficacy of SIRT for intrahepatic cholangiocarcinoma (NCT02807181), with results pending.

Additional areas of interest for interstitial brachytherapy include skin, rectal, and anal malignancies. For rectal adenocarcinoma patients with tumors smaller than 3 cm, brachytherapy boost with neoadjuvant chemoradiotherapy is an established treatment option based on randomized data showing that 3-year organ preservation rate was improved compared to external beam radiotherapy with neoadjuvant chemoradiotherapy, adding to the growing literature that intensified chemoradiation treatment can be a reasonable alternative for patients who seek alternatives to surgery [54]. Evaluation of the feasibility to deliver Diffusing Alpha-emitter Radiation Therapy (DaRT) to treat malignant skin and superficial soft tissue tumors in a single-institution pilot study is a novel direction of research to expand clinical indications for brachytherapy (NCT04377360). Likewise, early phase trials of high dose rate brachytherapy with concurrent chemotherapy aim to evaluate the safety profiles of dose escalation (NCT02199236) and clinical response (NCT01226979) anal and rectal cancer.

Among non-malignant diseases, we observed several completed or ongoing trials that investigated the use of strontium-90 brachytherapy to treat polyploid choroidal vasculopathy (NCT05251636) as well as treat (NCT02988895, NCT01006538) and reduce the burden of treatment (NCT01006538) for age-related macular degeneration. In addition, evaluation of brachytherapy safety and effectiveness to reduce recurrent coronary restenosis in coronary artery disease (NCT00714545, NCT00287573, NCT00180583) remains an area of research interest. We are hopeful that increasing collaboration between academic and industry partners will continue to develop novel applications for brachytherapy.

## Conclusion

The number of both single and multi-arm clinical trials including brachytherapy registered in ClinicalTrials.gov is increasing, suggesting continued research interest into brachytherapy applications. Clinical trials including brachytherapy were commonly Phase 2 and 3 trials, mostly evaluated in prostate, cervix, liver, and breast indications, and funded by academic sources.

Further research to design clinical trials including brachytherapy can increase the interest and evidence-based utilization of brachytherapy in clinical practice.

## Data Availability

All data generated or analysed during this study are included in this published article [and its supplementary information files].
